# Design and Creation of a Population-Wide Linkage Spine.

**DOI:** 10.23889/ijpds.v7i3.1870

**Published:** 2022-08-25

**Authors:** Elena Ougrinovski, Bronwyn Wilson

**Affiliations:** 1 Australian Institute of Health and Welfare (AIHW)

## Objectives

This paper describes the creation of a comprehensive, linked Australian population spine including name and address history. This spine was developed using four national datasets and linked to multiple State and Territory datasets. This enabled the creation of linkage maps which could then be used to produce de-identified linked datasets.

## Approach

Initially the spine was created using identifiers from Medicare Consumer Directory (MCD), Social Services data (DOMINO) and National Death Index (NDI). The COVID-19 vaccination program covering almost the entire Australian population provided the opportunity to add the Australian Immunisation Registry (AIR).

Probabilistic linkage was used to link MCD and DOMINO with linkage rates of 97.3%. MCD and NDI data were also linked probabilistically. Most AIR identifiers shared IDs with MCD and were linked deterministically with the remainder linked probabilistically.

## Results

Based on the linkage results, unique identifiers were created for everyone appearing in at least one of the four datasets. Only unlinked records with very incomplete information in NDI and AIR were excluded. All unique combinations of names and addresses for each individual were added to the combined spine. This allowed us to cover the data gaps of each dataset and create a comprehensive history not possible when using a single data source.

Linkage maps were created between all contributing data sources. State and Territory datasets were also linked to the spine using probabilistic linkage. These linkages were then reused for multiple projects.

## Conclusion and Relevance

Linkage to the combined spine increased linkage efficiency (a “Link once, use everywhere” approach) as well as increased linkage accuracy and a reduction in the transfer of personal information to provide a better service to the Australian research community.

